# Hydrology is a major influence on amphibian abundance in a large European floodplain

**DOI:** 10.1111/fwb.14104

**Published:** 2023-05-17

**Authors:** Andrew J. Hamer, István Czeglédi, Blanka Gál, Péter Sály, Zoltán Szalóky, Bálint Preiszner, Tibor Erős

**Affiliations:** ^1^ Balaton Limnological Research Institute Eötvös Loránd Research Network (ELKH) Tihany Hungary; ^2^ Institute of Aquatic Ecology, Centre for Ecological Research Budapest Hungary; ^3^ National Laboratory for Water Science and Water Security Balaton Limnological Research Institute Tihany Hungary; ^4^ National Laboratory for Water Science and Water Security Water Ecological Institute Budapest Hungary

**Keywords:** Danube River, habitat, metacommunities, river ecology, wetlands

## Abstract

1. River–floodplain ecosystems play a crucial role in connecting landscape patches through hydrological connectivity, but they are among the most threatened ecosystems. Floodplains provide important habitat for amphibians by connecting aquatic and terrestrial habitats. Modifications to floodplain hydrology can impact amphibian communities, yet few studies have examined amphibian metacommunities in floodplain wetlands.
2. In this study, we assessed patterns in amphibian breeding abundance in one of the largest floodplains of the Danube River, Hungary, relative to hydrological connectivity and multi‐scale variables at 30 waterbody sites. Our aim was to determine whether these patterns aligned with the pond‐permanence gradient hypothesis, where breeding amphibian abundance is predicted to be highest in ephemeral ponds without large predatory fish. We used Bayesian hierarchical modelling to estimate multi‐species abundance from repeated survey (count) data collected over one breeding season.
3. We detected the eggs and larvae of four amphibian species. The best model of abundance included covariates describing two principal component axes associated with waterbody hydrology and landscape composition within a 500‐m radius of a site. There was a positive relationship between mean community abundance at a site and hydrological disconnection from the main river channel; however, the common toad (*Bufo bufo*) was associated with hydrologically connected waterbodies. There was a positive relationship between mean community abundance and a high proportion of forest cover and low cover of agricultural land within a 500‐m radius around a site, although this relationship was clear for only two species. There was no support for models containing the number of large predatory fish species detected at a site.
4. Although our results showed that amphibian abundance declined with hydrological connectivity, based on model selection we could not ascribe this relationship to an increased number of large predatory fish species detected in waterbodies close to the main river channel. Differences in life history and habitat requirements are likely to have explained interspecific responses to hydrological connectivity. Our results underscore the importance of addressing amphibian abundance at multiple spatial scales in floodplain wetlands, as landscape composition partly explained patterns in abundance.
5. Application of multi‐species abundance modelling allowed us to investigate environmental relationships for common and infrequently detected species. Habitat restoration programmes in floodplains should provide waterbodies disconnected from main river channels as potential amphibian breeding sites and protect or restore forest as terrestrial habitat.

## 1 INTRODUCTION

River–floodplain ecosystems are crucial for maintaining high regional biodiversity and play an important role in linking various landscape patches through hydrological connectivity (Amoros & Bornette, 2002; Larsen et al., 2019; Tockner et al., 1998). Furthermore, flood events shape and maintain a complex mosaic of riparian landforms and biotic communities, while riparian corridors facilitate the migration of biota and enhance ecological connectivity (Naiman & Décamps, 1997; Ward, 1998). Despite their crucial importance for biodiversity, floodplain landscapes are among the most endangered ecosystems globally (Habersack et al., 2016; Tockner & Stanford, 2002; Ward et al., 1999); for example, more than 90% of the former floodplains in Europe have either disappeared or no longer function effectively (Tockner et al., 2006). Hydro‐morphological alterations resulting from engineering works have dissociated most large river channels from their previously integrated floodplains, which has resulted in substantial changes in the composition of ecological communities (e.g., Reckendorfer et al., 2006; Schiemer et al., 1999).

Metacommunity theory is a relevant conceptual framework for understanding the organisation of freshwater communities, where connectivity and environmental heterogeneity may differ between major aquatic systems (Heino et al., 2015). For instance, in river–floodplain landscapes aquatic connectivity may induce mass effects, where high dispersal rates homogenise communities at neighbouring localities, regardless of their abiotic and biotic environmental conditions, whereas communities at isolated waterbodies may be structured by dispersal limitation (Heino et al., 2015). In other instances, species‐sorting may prevail, in which species are “filtered” by environmental factors. Flood frequency and hydrological connectivity are the main factors controlling the patterns of local communities and, therefore, patterns in floodplain metacommunities (Fernandes et al., 2014; Larsen et al., 2019). There are far fewer floodplain metacommunity studies than those conducted at the river network scale, despite serving as ideal model systems for testing hypotheses relating to changes in local communities in response to dispersal and environmental gradients (Heino et al., 2015).

Amphibian populations have been declining in abundance globally for several decades owing to multiple environmental stressors leading to widespread losses in metapopulations (Campbell Grant et al., 2016). Introduced fish have caused massive losses of amphibian breeding sites as they are efficient predators of larval amphibians in freshwater ecosystems (Falaschi et al., 2020; Knapp & Matthews, 2000). Enhancement of hydrological connectivity in restoration programmes often aims to increase fish dispersal to the detriment of many amphibian species because of increased predation rates, especially in waterbodies that were fish‐free before restoration (Tockner et al., 1998). Floodplains provide crucial aquatic and terrestrial habitats for many amphibian species to complete their complex life cycles (Henning & Schirato, 2006; Joly & Morand, 1994), and amphibians provide numerous ecological benefits to floodplain ecosystems (McGinness et al., 2014; Ocock et al., 2016), but modifications to floodplain hydrology and invasive fish can lead to population declines (Holgerson et al., 2019). Despite the recognition of the importance of amphibian communities in floodplain ponds (Jansen & Healey, 2003; Joly & Morand, 1994; Morand & Joly, 1995), there is currently insufficient knowledge of amphibian ecology in floodplain wetland systems compared to other biota (Ocock et al., 2016; Tockner et al., 2006), and amphibian occupancy patterns in floodplain wetlands have seldom been examined (Holgerson et al., 2019).

Here, we examined patterns in the abundance of amphibian communities across a river–floodplain landscape along a section of the Danube River in southern Hungary. The aim was to determine community and species' responses to hydrological connectivity and a range of landscape‐ and local‐scale variables postulated to influence amphibian abundance. We assessed breeding abundance, including counts of egg masses and larvae (including metamorphosing individuals), because of the important relationship between reproduction and population viability (Van Horne, 1983), and because the early life stages of amphibians are strongly affected by aquatic predators and hydroperiod (Berven, 1990; Semlitsch et al., 1996). We examined amphibian responses to wetland hydrology to test whether they aligned with the pond‐permanence gradient hypothesis, where large fish predators are predicted to be most abundant in permanent freshwater ponds while typically absent in ephemeral ponds (Wellborn et al., 1996). For example, on floodplains amphibians are often restricted in their occurrence to isolated waterbodies, free of fish (Tockner et al., 1998). According to theoretical models of metacommunity organisation in floodplain wetlands (Heino et al., 2015) and freshwater ponds (e.g., Wellborn et al., 1996; Werner et al., 2007), we predicted there would be an increase in amphibian abundance across a gradient of hydrological connectivity, with highest abundance at sites most disconnected from the main river channel. We also evaluated breeding abundance relative to the landscape surrounding ponds, as amphibians require aquatic and terrestrial habitats interconnected by dispersal to maintain complex life cycles (Semlitsch, 2002). Based on our modelling predictions, we provide recommendations for conserving amphibian communities in floodplain habitats.

## 2 METHODS

### 2.1 Study area

**FIGURE 1:**
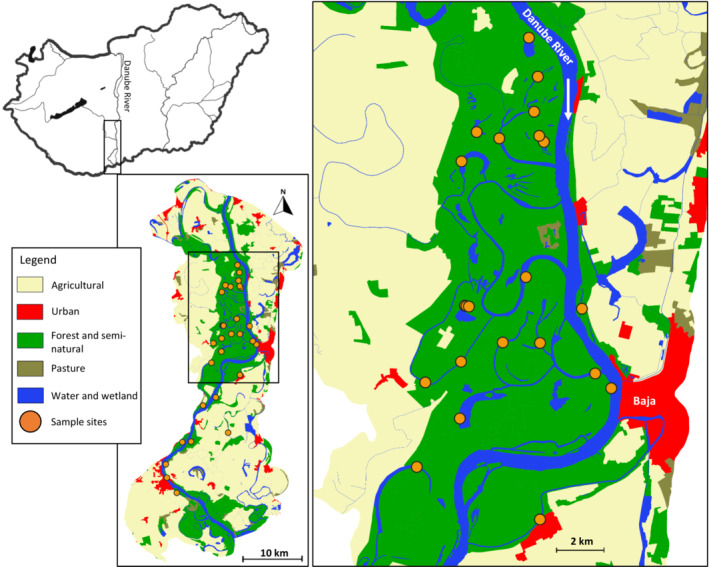
Map of the study area in the Middle Danube floodplain, southern Hungary. The enlarged inset depicts sites distributed across the Gemenc floodplain and highlights the diversity of waterbody types surveyed.

The study was conducted within the Danube River floodplain, situated in the Middle Danube, Southern Hungary (Figure 1). The study area includes the Gemenc floodplain which is one of the largest floodplain areas in Europe (c. 180 km^2^; Schöll & Devetter, 2013). Although the Danube River has been channelised, confined by levees, impounded and polluted (Habersack et al., 2016; Hohensinner et al., 2004), this section of the Danube floodplain remains the largest functioning floodplain in the Middle Danube (Funk et al., 2021; Hein et al., 2016). It contains diverse floodplain habitat types (e.g., side arms, backwaters and oxbows with different degrees of hydrological surface connectivity; see Hohensinner et al., 2004). Most of the floodplain is protected under the Danube‐Dráva National Park.

### 2.2 Study sites

Waterbody sites were selected along a hydrological gradient of pulsing and disturbance within the schematic representation of metacommunity organisation (see Heino et al., 2015). Sites included sections of the main channel of the Danube River, and a range of waterbody types including sidearms, backwaters, oxbow lakes, and floodplain ponds and marshes, so that the 30 sites were representative of the heterogeneity in hydrology and habitat structure present across the floodplain landscape. Sites were arranged into spatial clusters based on distance to the nearest neighbour site; sites within the same cluster were generally surveyed on the same date; and the order in which clusters were surveyed was randomised.

### 2.3 Amphibian surveys

Three surveys were conducted at the 30 sites over a single breeding season in spring and summer (Survey 1: March 2021; Survey 2: May 2021; Survey 3: August 2021), corresponding with the breeding season of amphibian species recorded in the region (Berninghausen & Berninghausen, 2001). Repeated surveys over the full breeding season were undertaken to account for potentially high variability in egg and larval abundance at sites. Surveys comprised dip‐netting and visual surveys for the egg masses and larvae of amphibians during the daytime conducted by two to three people. Dip‐netting was conducted at waterbodies with sufficient water levels (water depth >5 cm) using a net design appropriate for the safe capture of amphibians and their larvae (300‐mm‐wide frame, 350 mm deep, 2‐mm mesh size). The number of net sweeps was standardised to be proportional to the sampling area. For the main channel and wide tributary arms, we selected 500‐m transects and conducted 30 sweeps with a dip‐net within an area of 30 m × 30 m every 100 m (i.e., six sampling plots; *n* = 180 sweeps). For other waterbodies (e.g., ponds, oxbow lakes) we did one dip‐net sweep per 25 m^2^ of water surface area (Shulse et al., 2010), with a minimum of two sweeps and a maximum capped at 180 sweeps. We adjusted the number of sweeps at a site according to water levels; for example, by decreasing the number of sweeps when low water levels reduced water surface area. Dip‐net sweeps were approximately 1.5 m in length and were performed by two persons in all available microhabitat types (e.g., open water, aquatic vegetation) to target the aquatic microhabitat preferences of amphibians (Shaffer et al., 1994).

Amphibians were identified and counted upon capture and then released, with the distance between the release location and the next dip‐net sweep being >5 m to avoid double‐counting individuals. In some smaller waterbodies amphibian larvae caught in dip‐nets were held temporarily in a plastic bucket, and then identified, counted and released unmarked. We counted metamorphosing amphibians that were observed while dip‐netting using visual encounter surveys (Crump & Scott Jr., 1994). The number of egg masses of the agile frog (*Rana dalmatina*) observed while dip‐netting were counted at sites as a proxy for the number of reproductively‐mature females, assuming that each female lays only one egg mass per year (Hartel et al., 2009). Egg mass counts were conducted to coincide with the peak spawning period in March and were included in the total amphibian counts. Egg masses of *R. dalmatina* can be reliably identified at this time and occur as individual clumps in ponds, predominantly around the shoreline (Băncilă et al., 2016). The count of all macroscopic organisms (e.g., fish) captured in dip‐nets was recorded. Water temperature was recorded at 1 m from the waterline at the start of each survey. Water levels were recorded during each survey as a percentage of the full water‐holding capacity of a site.

Amphibians were identified to species using Berninghausen and Berninghausen (2001). Taxonomy follows (Speybroeck et al., 2020). Green frogs (*Pelophylax lessonae*, *P. kl. esculenta* and *P. ridibunda*) were consolidated within the *Pelophylax* spp. complex. Frog and toad larvae were defined as individuals within Gosner development stages 25 (small tadpoles large enough to be reliably identified) to 42–44 (metamorphosing tadpoles with front and hind limbs; Gosner, 1960). Standard hygiene protocols to minimise the risk of spreading the amphibian chytrid fungus (*Batrachochytrium dendrobatidis* and *B. salamandrivorans*) were followed when conducting fieldwork (Phillott et al., 2010).

### 2.4 Local‐scale habitat variables

The area of waterbody surface sampled at each site was calculated using a Geographical Information System (QGIS version 3.16; QGIS Development Team, 2020) and Google Earth Pro (Google Earth version 7.3.6.9345). At isolated ponds, this area corresponded to the area of the entire pond surface, but elsewhere area corresponded only to the waterbody sections that were sampled (e.g., along the transects of the main channel and tributaries, or within sample plots of oxbow lakes). Mean sampling area was 11,031 m^2^ (*SD* 20,505, range 284–114,069 m^2^). The distance between the respective edges of sites (i.e., nearest neighbour distance) was measured using Google Earth Pro (mean 1,689 m, *SD* 1,544, range 29–6403 m).

Habitat structure was recorded at each site during surveys 2 and 3 (May and August), comprising estimates of the percentage cover of emergent, submerged and floating vegetation, surface algae, canopy cover and woody debris; and percentage of the waterbody perimeter ± 2 m from the shoreline comprised of woody vegetation (e.g., trees), fringing vegetation (macrophytes, herbs, grasses) and rip‐rap/concrete. Habitat structure was recorded in each sampling plot along site transects (i.e., six plots) and the mean taken, or was estimated for the entire pond or sampling plot at non‐transect sites.

Water chemistry was recorded during surveys 2 and 3 from two sampling points at each site ~1 m from the margin of the water surface and at a depth of 5–10 cm using a handheld electronic meter (Aquaprobe®; Aquaread Ltd). Samples at transect sites were measured within the start and end plots; elsewhere, samples were taken from two points at respective ends of the waterbody. Measured parameters included: pH, electrical conductivity (μS/cm), total dissolved solids (TDS; mg/L), salinity (PSU; practical salinity units), dissolved oxygen (mg/L) and temperature (°C). No measurements were taken at waterbodies with low water levels (<15 cm) because the Aquaprobe® could not be fully immersed. Water chemistry values at these sites were replaced by mean values in the analyses.

The presence of fish was determined from dip‐netting (smaller species) and visual observations. We also used environmental DNA (eDNA) techniques at sites to detect fish as part of a study on fish communities (see Czeglédi et al., 2021). We used the number of large fish species detected at a site (i.e., species richness), including only species >1,000 g in mean weight that are carnivorous/piscivorous (Froese & Pauly, 2022), thereby excluding large omnivorous species (e.g., *Carassius* sp.). Larger predatory fish were expected to have a greater overall impact on amphibian reproduction at a site than smaller fish (Hecnar & M'Closkey, 1997; Kloskowski, 2009).

Hydrological connectivity was defined as the average annual duration during a year (% of days) that a site was connected to the main channel of the Danube (Funk et al., 2013; Reckendorfer et al., 2006). It was calculated using data on the stage–discharge relationships of the Danube from the period 2000–2016, the discharge frequency distribution of the river, and the stage at which the water flows into a waterbody site using the Adaptive Hydraulics (AdH; https://www.erdc.usace.army.mil/) modelling system (Molnár, 2021). A digital elevation model (DEM) was used to define water inflows and calculate mean water depth of the waterbodies. Field visits in 2021 were used to validate model performance and refine water inflow and water depth data for some sites if required. Hydroperiod was determined by recording water levels at sites during each survey. Sites which were not observed to dry out during surveys were classed as permanent, whereas ephemeral sites dried out completely.

### 2.5 Landscape‐scale variables

The proportion covers of the following landscape variables were calculated using layers of the CORINE Land Cover 2018 database (European Environmental Agency 2020, http://www.eea.europa.eu) in GIS within a 500‐m radius around the centroid of each waterbody site: (1) forest and semi‐natural land; (2) agricultural land; (3) pasture; (4) urban land; and (5) water and wetland surface. A 500‐m radius was considered sufficient to encompass the mean dispersal distances of amphibian species expected to occur in the study area (Kovar et al., 2009; Smith & Green, 2005; Vos & Stumpel, 1995).

### 2.6 Principal components analysis

There were inter‐correlations among the habitat variables (see Tables S1–S4) which were assigned into the categories: (1) habitat structure; (2) water chemistry; (3) hydrology; and (4) landscape. Accordingly, principal components analysis (PCA) was used to ordinate habitat variables and reduce the number of variables (Legendre & Legendre, 2012). Separate PCAs were conducted for each habitat category using the program R (version 4.1.2; R Core Team, 2019). Component scores from each of the first PC axes subsequently were used as site values for each covariate in modelling amphibian abundance. Sampling area, nearest neighbour distance and large fish species richness were excluded from the PCAs. Habitat variables were ordinated onto PC axes to represent the complex gradients of habitat conditions and hydrological connectivity that characterises riverscapes (Amoros & Bornette, 2002). There were no strong correlations among the four PCs, sampling area and nearest neighbour distance (Table S5). There was a strong correlation between the hydrology component scores at sites and large fish species richness (*r* = −0.704; Table S5).

### 2.7 Multi‐species abundance modelling

Multi‐species abundance models (MSAM) were used to assess relationships between the breeding abundance of amphibians at sites and the covariates. These models enable abundance to be estimated from repeated count surveys while adjusting for imperfect detection of individuals (Royle, 2004; Royle et al., 2005, 2007). Individual species models are linked into a collective hierarchical model so that they represent community‐level responses to environmental covariates, thereby increasing the precision of parameter estimates for rarely‐observed species by considering each within the context of the broader community and borrowing strength from more abundant species (Dorazio et al., 2006; Kéry & Royle, 2008; Zipkin et al., 2009).

Counts of amphibian egg masses and larvae were summed at each site for each survey and subsequently used in MSAM, which were constructed from a series of individual *N*‐mixture models using the original formulation of Royle (2004) and Royle et al. (2005). The first level of the model (sub‐model) assumed true but imperfectly observed abundance, where the abundance of species *i* at site *j*, *N*_*ij*_, is a Poisson random variable:

$$N_{\mathrm{ij}}\sim \text{Poisson}\;\left(\lambda_{\mathrm{ij}}\right)$$

where *λ*_*ij*_ is the expected (or mean) abundance (Royle et al., 2005). Overdispersion in count data is common and can bias parameter and abundance estimates in *N*‐mixture models (Knape et al., 2018; Link et al., 2018). A random effects term for overdispersion and unexplained variation in abundance arising from repeated counts among sites (*ε*_*j*_) therefore was included in models (Kéry et al., 2009). Mean abundance was expressed as a log‐linear function of site‐level covariates in nine models (Table 2):

$$\log\;\left(\lambda_{\mathrm{ij}}\right)=\beta_{0i}+\beta_{1i}\;\left({\text{Area}}_{j}\right)+\beta_{2i}\;\left(x_{1j}\right)+\beta_{3i}\;\left(x_{2j}\right)+\varepsilon_{j}$$

where *x*_1*j*_ and *x*_2*j*_ were site covariates. Area and nearest neighbour distance were log_10_(*x*)‐transformed before analysis. Area was included in all models to account for variation in estimated abundance as a consequence of sampling area. Each model had a maximum of three covariates given the recommendation in linear models of a minimum *n/k* of 10, where *n* is the number of sites and *k* is the number of estimated parameters (Harrison et al., 2018).

The probability of detection was modelled using the number of days since 1 February (Days) and water temperature (Temp). Days accounts for variation in detection since the beginning of the breeding season – thus, interspecific differences in breeding phenology (Berninghausen & Berninghausen, 2001). Amphibian activity and hence detectability is likely to increase with water temperature (Petitot et al., 2014). Detection was modelled as a binomial process:

$$C_{\mathrm{ijk}}\sim \text{Binomial}\;\left(p_{\mathrm{ijk}},N_{\mathrm{ij}}\right)$$

where *C*_*ijk*_ is the number of detected individuals (i.e., count) of species *i* at site *j* on survey *k*, and *p*_*ijk*_ is the probability of detecting a single individual of species *i* at site *j* on survey *k* (Royle et al., 2005). Detection probability was expressed as a logit‐link function of the two survey‐specific covariates in each model:

$$\text{logit}\;\left(p_{\mathrm{ijk}}\right)=\beta_{0i}+\beta_{1i}\;\left({\text{Days}}_{\mathrm{jk}}\right)+\beta_{2i}\;\left({\text{Temp}}_{\mathrm{jk}}\right)$$

Site covariates in abundance and detection sub‐models were standardised as *z* scores (mean 0; *SD* 1), which allowed direct comparison of model coefficients so that the relative importance of each covariate could be determined according to the magnitude of the estimated model coefficient (Schielzeth, 2010).

Community‐level hyper‐parameters (*μ*) were treated as random effects that governed species‐level parameters (Zipkin et al., 2009). Community summaries and model parameters were estimated using Bayesian inference with priors for the hyper‐parameters drawn from a normal distribution; *N* (−1, 5) for intercept terms (*β*_0*i*_), *N* (1, 5) for *β*_1*i*_, *β*_2*i*_, *β*_3*i*_ (see Hamer et al., 2021). Hyper‐parameters for precision (*σ*) were drawn from a uniform distribution (*U* [0.01, 0.5]); the overdispersion term also was modelled using a uniform prior (*U* [0, 1]). Species were assumed to have broadly similar responses to covariates; thus, species responses were drawn from a common distribution where the species have similar ecological relatedness (Pacifici et al., 2014).

The nine models were compared using the Watanabe–Akaike information criterion (WAIC; Watanabe, 2010), computed by the methods of Gelman et al. (2014). The WAIC is a fully Bayesian method, based on the posterior predictive distribution of mean parameter estimates that is valid for hierarchical models (Hooten & Hobbs, 2015). The best‐supported model had the lowest WAIC value (Burnham & Anderson, 2002). Bayesian *p*‐values were used to assess model fit by calculating the Freeman–Tukey fit statistic (see Stolen et al., 2019). Values close to 0.5 indicate acceptable model fit whereas *p* <0.1 indicates a potential lack‐of‐fit (Conn et al., 2018; Gelman, 2013).

The mean and *SD* of the model coefficients derived from the best‐supported model are presented, and the 2.5th and 97.5th percentiles of the posterior distribution, which represents a 95% Bayesian credible interval (BCI). Parameter estimates of covariates with a BCI that did not overlap zero were clearly important, whereas estimates with a BCI overlapping zero had greater uncertainty and were considered to be less important. However, some minor overlap of the BCI with zero was tolerated in inferring relationships (see Cumming & Finch, 2005). Covariates with smaller standard deviation on hyper‐parameters (*σ*_*λ*_) were considered to have similar effects across all amphibian species; larger *σ*_*λ*_ values indicated dissimilar effects.

Modelling was performed using the software program JAGS (version 4.3.0; Plummer, 2017) called via the R2jags package (Su & Yajima, 2015) from program R. Each model was run using three replicate Markov chain Monte Carlo (MCMC) iterations to generate 800,000 samples from the posterior distribution of each model after discarding a “burn‐in” of 50,000 samples, with a thinning rate of 5. Convergence of the Markov chains was checked using the Brooks–Gelman–Rubin statistic $\left(\hat{R}\right)$; acceptable convergence was achieved when $\hat{R}$ < 1.1 (Brooks & Gelman, 1998).

## 3 RESULTS

### 3.1 Amphibian surveys

The eggs and larvae of a total of four amphibian species were detected during the surveys (Table 1). The egg masses of *R. dalmatina* were detected only in Survey 1 at a total of 20 sites where the mean number of egg masses was 50.5 (*SD* 70.6, range 1–259). No other species were detected in Survey 1 and amphibian eggs were not detected in surveys 2 and 3. The larvae of the common toad (*Bufo bufo*) were detected at 11 sites (Table 1), where it was numerically the most abundant species (mean no. larvae 458.4, *SD* 752.6, range 1–2264). Larvae of the green frogs (*Pelophylax* spp. complex) were detected at six sites while larvae of the European tree frog (*Hyla arborea*) and *R. dalmatina* were each detected at four sites (Table 1).

**TABLE 1:** Detection of the eggs and larvae of four amphibian species, hydrological parameters and large fish species richness at 30 waterbodies in the Middle Danube floodplain, southern Hungary.

Site no.	Species^a^				Hydrology^b^		Large fish^c^
	Bufbuf	Hylarb	Pelcom	Randal	Hydro	%Conn	No. of species
1	●	○	○	▲●	1	0	2
2	○	○	○	▲	1	0	0
3	●	○	●	▲	1	2	3
4	○	○	○	○	1	80	6
5	○	○	○	▲	0	15	5
6	●	○	○	▲	1	45	6
7	○	○	○	▲	1	50	8
8	●	○	●	▲	1	25	4
9	○	○	○	▲●	1	10	4
10	●	○	○	○	1	25	4
11	○	○	○	○	1	100	7
12	○	○	○	○	1	100	6
13	○	○	○	○	1	90	6
14	○	○	○	▲	1	1.5	1
15	○	○	○	▲	1	20	5
16	○	○	○	▲	0	20	4
17	○	○	○	○	1	48	5
18	●	●	○	▲●	1	5	6
19	○	○	○	▲	0	5	0
20	●	○	○	▲	1	20	5
21	○	●	●	▲	0	5	0
22	○	●	○	▲	0	5	0
23	○	○	○	○	1	70	7
24	○	○	○	○	1	0	5
25	●	●	●	▲●	1	8	1
26	●	○	○	○	1	90	6
27	●	○	●	▲	1	90	8
28	○	○	●	▲	1	18	7
29	●	○	○	▲	1	50	6
30	○	○	○	○	1	90	7

*Note*: ▲, egg masses detected; ●, larvae detected; ○, no eggs or larvae detected.

^a^ Species: Bufbuf = *Bufo bufo*; Hylarb = *Hyla arborea*; Pelcom = *Pelophylax* spp. complex; Randal = *Rana dalmatina*.

^b^ Hydro = Hydroperiod (1 = permanent; 0 = ephemeral); %Conn = % of days connected to the main channel of the Danube River.

^c^ Large fish = number of large predatory fish species detected (see Table S6 for list of species).

### 3.2 Hydrological conditions

Five ephemeral sites were dry in Survey 2 (Table 1). There was a major flood event at the Danube River in July 2021 immediately before Survey 3, with floodwaters refilling ephemeral sites and raising water levels at permanent sites. The height of the flood level (2,175 cm) had a mean recurrence frequency of every 3–5 years, based on historical flood data. The larvae of *H. arborea* were detected in Survey 3 at two of these refilled ephemeral sites, whereas *Pelophylax* spp. larvae were detected at one refilled site. The percentage of days connected to the main channel ranged from 0% at two ponds in riparian forest and a site in agricultural land (with no flooding), to 100% at two sites situated along the main river channel (mean 36.3%, *SD* 35.7; Table 1).

### 3.3 Predatory fish

A total of 11 large predatory fish species were detected (Table S6). The number of species detected at sites ranged from 0 to 8 (mean 4.5, *SD* 2.5; Table 1), with no detections of large fish at four sites of which three were ephemeral waterbodies. Northern pike (*Esox lucius*) and asp (*Leuciscus aspius*) were detected at 23 (0.77) and 21 sites (0.70), respectively. Four species were each detected only at a single site. There was a strong correlation between the number of large predatory fish species at a site and the percentage of days in a year that a site was connected to the main channel of the Danube River (*r* = 0.703). Six small predatory fish species also were detected at sites (Table S6). There was a strong positive correlation between the number of small and large predatory fish species at a site (*r* = 0.845).

### 3.4 Principal component analyses

The first principal component (PC 1) from the hydrology PCA revealed that the first axis (PC 1) explained 61.9% of the variation, and was negatively correlated with permanence, water depth and % days connected (Table S7). The first axis (PC 1) from the habitat structure PCA explained 28.0% of the variation among the variables, with % fringing and % emergent vegetation (positive factor loadings) and % woody debris (negative factor loading) most strongly correlated with this axis (Table S8). Principal component 1 of the water chemistry PCA explained 49.7% of the variation and was positively associated with electrical conductivity, salinity, total dissolved solids and temperature (Table S9). Principal component 1 of the landscape PCA explained 51.1% of the variation, with a strong negative association with proportion forest cover within a 500‐m radius and positive associations with the proportion cover of agricultural land and pasture (Table S10).

### 3.5 Model selection and fit

The best‐ranked model included Area, Hydrology and Landscape (WAIC = 17,668; Model 1, Table 2). There was no support for models containing nearest neighbour distance, water chemistry parameters or large fish species richness. Model 1 had acceptable model fit (Bayesian *p* = 0.120) and was used to derive estimates of mean breeding abundance and probabilities of detection.

**TABLE 2:** Watanabe–Akaike information criterion (WAIC) values for nine multi‐species abundance models of breeding amphibian communities across 30 waterbodies in the Middle Danube floodplain, southern Hungary.

Model^a^			Covariates^b^		WAIC
**1**	**Intercept**	**Area**	**Hydrology**	**Landscape**	**17,668**
2	Intercept	Area	Hydrology	Water	22,361
3	Intercept	Area	Landscape	Water	25,159
4	Intercept	Area	Landscape	Distance	34,640
5	Intercept	Area	Hydrology	Habitat	38,534
6	Intercept	Area	Landscape	Fish	38,987
7	Intercept	Area	Fish	Water	43,685
8	Intercept	Area	Fish	Habitat	51,889
9	Intercept	Area	Landscape	Habitat	57,657

^a^ Best‐ranked model (smallest WAIC) is shown in bold.

^b^ Area = sampled waterbody area; Hydrology = Principal Component (PC) 1, describing a gradient from deep permanent waterbodies with a high % of days connected to the main river channel (negative values: connected waterbodies) to ephemeral shallow waterbodies with a low % of days connected (positive values: disconnected/isolated waterbodies); Landscape = PC 1 describing a gradient from waterbodies surrounded by a high % cover of forest and semi‐natural land within a 500‐m radius (negative values) to waterbodies surrounded by a high % cover of agricultural land and pasture (positive values); Water = PC 1 describing a gradient from waterbodies with high pH (negative values) to waterbodies with high electrical conductivity, salinity and total dissolved solids (positive values); Distance = nearest neighbour distance; Habitat = PC 1, describing a gradient from waterbodies with a high % cover of woody debris (negative values) to waterbodies with a high % cover of fringing, floating and emergent vegetation (positive values); Fish = number of species of large predatory fish at a site.

### 3.6 Mean community abundance

**FIGURE 2:**
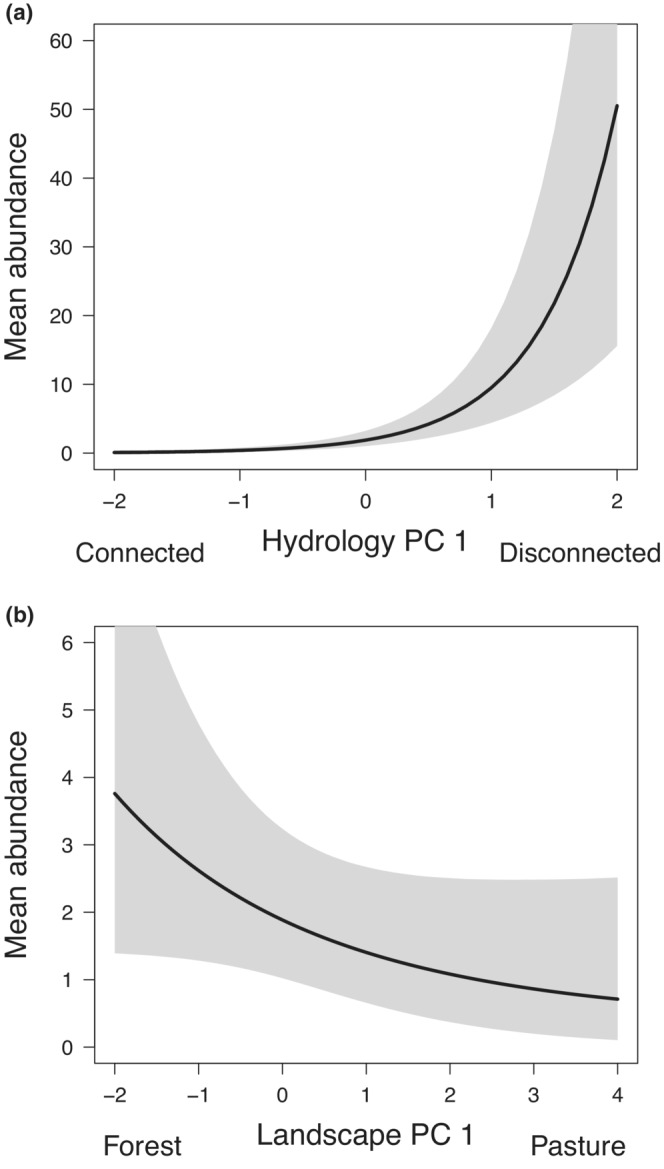
Mean estimates of breeding abundance (shaded areas are 95% Bayesian credible intervals) across the amphibian community versus principal component (PC) scores for the first axes of the (a) hydrology PC analysis (PCA) and (b) landscape PCA. See Table 2 for a description of the PC scores. Note the different numerical scales for mean abundance.

There was a positive relationship between mean community abundance and Hydrology PC 1 (*μ*_*λ*2_ = 2.175, 95% BCI 1.560–2.788; Table 3). Mean community abundance (i.e., mean number of egg masses and larvae) was predicted to increase from 0.09 at the sites most strongly associated with negative scores for PC 1 (sites with greatest hydrological connectivity), to 50.52 at the sites most strongly associated with positive scores of PC 1 (sites with greatest disconnection or isolation from the main channel; Figure 2a). There was relatively low variability in Hydrology PC 1 across the six species relative to the mean value of the coefficient and narrow credible intervals (*σ*_*λ*2_ = 0.495, 95% BCI 0.483–0.500; Table 3), indicating similar responses to hydrological connectivity within the community.

**TABLE 3:** Summary of community‐level hyper‐parameters for abundance (*λ*) and detection (*β*) of egg masses and larvae of four amphibian species.

Hyper‐parameter^a^	Covariates^b^	Mean	*SD*	2.5th	97.5th
*μ*_*λ*0_	Intercept	0.591	0.294	0.022	1.175
*σ*_*λ*0_	Intercept	0.491	0.009	0.466	0.500
***μ***_***λ*1**_	**Area**	**0.847**	**0.247**	**0.361**	**1.331**
*σ*_*λ*1_	Area	0.355	0.099	0.135	0.494
***μ***_***λ*2**_	**Hydrology**	**2.175**	**0.313**	**1.560**	**2.788**
*σ*_*λ*2_	Hydrology	0.495	0.005	0.483	0.500
*μ*_*λ*3_	Landscape	−0.504	0.294	−1.081	0.072
*σ*_*λ*3_	Landscape	0.472	0.025	0.405	0.499
*μ*_*β*0_	Intercept	−4.183	0.274	−4.721	−3.649
*σ*_*β*0_	Intercept	0.460	0.044	0.343	0.499
*μ*_*β*1_	Days	0.129	0.220	−0.299	0.562
*σ*_*β*1_	Days	0.470	0.026	0.402	0.499
***μ***_***β*2**_	**Temp**	**1.361**	**0.226**	**0.916**	**1.804**
*σ*_*β*2_	Temp	0.493	0.007	0.475	0.500

*Note*: Estimates include 95% Bayesian credible intervals (2.5th and 97.5th percentiles of the posterior distribution). Important relationships for hyper‐parameters of the covariates are where the 95% BCI does not overlap zero (highlighted in bold, except intercept coefficients).

*Abbreviations*: *μ*, mean community response; *σ*, standard deviation in response to the covariate across species; Days, number of days since 1 February 2021; Temp, water temperature.

^a^ Hyper‐parameter estimates were extracted from Model 1 (see Table 2).

^b^ See Table 2 for a description of the covariates used to estimate abundance (*λ*).

There was a negative relationship between mean community abundance and Landscape PC 1, although the 95% BCI overlapped zero slightly (*μ*_*λ*3_ = −0.504, 95% BCI −1.081 to 0.072; Table 3). Mean community abundance was predicted to decrease from 3.76 at the sites most strongly associated with negative scores for PC 1 (sites with a high proportion of forest cover within a 500‐m radius), to 0.71 at the sites most strongly associated with positive scores for PC 1 (sites with a high proportion cover of agricultural land and pasture; Figure 2b). There was relatively low variability in Landscape PC 1 relative to the mean coefficient and narrow credible intervals (*σ*_*λ*3_ = 0.472, 95% BCI 0.405–0.499; Table 3), indicating similar responses among species to landscape composition.

There was a positive relationship between mean community abundance and area sampled (*μ*_*λ*1_ = 0.847, 95% BCI 0.361–1.331; Table 3).

### 3.7 Individual species abundance

**FIGURE 3:**
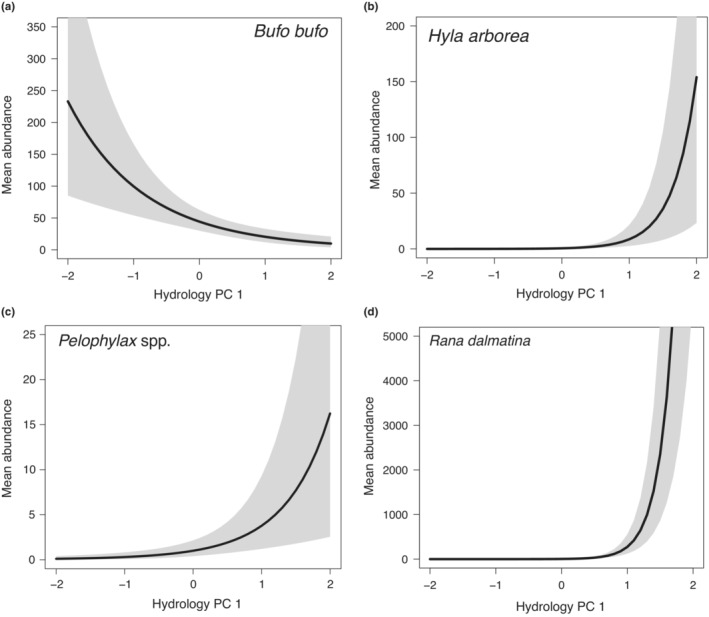
Mean estimates of the breeding abundance of four amphibian species versus principal components (PC) scores for the first axis of the hydrology PC analysis (PC 1). Note the different numerical scales for mean abundance.

There were positive relationships between mean abundance and Hydrology PC 1 for three species (Figure 3), with the strongest relationship for *R. dalmatina* (*λ*_2_ = 5.775, 95% BCI 5.078–6.473) and weakest relationship for the *Pelophylax* spp. complex (*λ*_2_ = 1.749, 95% BCI 0.773–2.730; Table 4; Figure 3). There was a negative relationship between mean *B. bufo* abundance and Hydrology PC 1 (*λ*_2_ = −1.074, 95% BCI −1.606 to −0.537; Table 4; Figure 3).

**TABLE 4:** Summary of species‐specific estimates for abundance (*λ*) and detection (*β*) covariates for egg masses and larvae of four amphibian species.

Species	Species‐specific parameter^a^		Mean	*SD*	2.5th	97.5th
*Bufo bufo* (Family: Bufonidae)	*λ*_0_	Intercept	3.771	0.189	3.401	4.138
	***λ***_**1**_	**Area**	**0.936**	**0.208**	**0.526**	**1.341**
	***λ***_**2**_	**Hydrology**	**−1.074**	**0.273**	**−1.606**	**−0.537**
	*λ*_3_	Landscape	0.174	0.234	−0.290	0.628
	*β*_0_	Intercept	−4.835	0.103	−5.040	−4.637
	***β***_**1**_	**Days**	**−1.243**	**0.066**	**−1.375**	**−1.114**
	***β***_**2**_	**Temp**	**4.955**	**0.117**	**4.730**	**5.189**
*Hyla arborea* (Family: Hylidae)	*λ*_0_	Intercept	−0.698	0.452	−1.567	0.199
	*λ*_1_	Area	0.338	0.325	−0.305	0.965
	***λ***_**2**_	**Hydrology**	**3.691**	**0.510**	**2.688**	**4.678**
	*λ*_3_	Landscape	−0.402	0.480	−1.337	0.543
	*β*_0_	Intercept	−5.392	0.426	−6.240	−4.573
	***β***_**1**_	**Days**	**1.217**	**0.231**	**0.776**	**1.682**
	*β*_2_	Temp	0.261	0.255	−0.238	0.761
*Pelophylax* spp. complex (Family: Ranidae)	*λ*_0_	Intercept	−0.092	0.426	−0.917	0.769
	***λ***_**1**_	**Area**	**1.232**	**0.263**	**0.713**	**1.747**
	***λ***_**2**_	**Hydrology**	**1.749**	**0.497**	**0.773**	**2.730**
	***λ***_**3**_	**Landscape**	**−1.330**	**0.402**	**−2.118**	**−0.542**
	*β*_0_	Intercept	−4.942	0.398	−5.730	−4.168
	***β***_**1**_	**Days**	**−0.623**	**0.198**	**−1.026**	**−0.248**
	***β***_**2**_	**Temp**	**0.901**	**0.185**	**0.541**	**1.267**
*Rana dalmatina* (Family: Ranidae)	*λ*_0_	Intercept	1.298	0.342	0.662	1.985
	***λ***_**1**_	**Area**	**0.777**	**0.214**	**0.356**	**1.196**
	***λ***_**2**_	**Hydrology**	**5.775**	**0.359**	**5.078**	**6.473**
	***λ***_**3**_	**Landscape**	**−2.137**	**0.316**	**−2.755**	**−1.508**
	*β*_0_	Intercept	−4.945	0.279	−5.484	−4.414
	***β***_**1**_	**Days**	**0.201**	**0.049**	**0.106**	**0.297**
	***β***_**2**_	**Temp**	**−0.235**	**0.045**	**−0.323**	**−0.147**

*Note*: Estimates include 95% Bayesian credible intervals (2.5th and 97.5th percentiles of the posterior distribution). Parameter estimates were extracted from Model 1 (see Table 2). Important relationships are where the 95% BCI does not overlap zero (highlighted in bold, except intercept coefficients).

^a^ See Tables 2 and 3 for descriptions of the covariates used to estimate abundance (*λ*) and detection (*β*), respectively.

There was a negative relationship between the mean abundance of *R. dalmatina* and Landscape PC 1 (*λ*_3_ = −2.137, 95% BCI −2.755 to −1.508; Table 4); thus, a strong association with sites surrounded by a high cover of forest. There also was a strong relationship between the mean abundance of *Pelophylax* spp. and a high cover of forest surrounding sites. There were no clear relationships between mean abundance of *B. bufo* and *H. arborea* and Landscape PC 1 (Table 4).

There were positive relationships between the mean abundances of *B. bufo*, *Pelophylax* spp. and *R. dalmatina*, and waterbody area sampled (Table 4).

### 3.8 Probability of individual detection

There was a positive relationship between the mean probability of individual detection across the community and water temperature (*μ*_*β*2_ = 1.361, 95% BCI 0.916–1.804; Table 3). There was no clear relationship between the mean probability of detection and the number of days elapsed since 1 February on a survey (Table 3). There were generally clear relationships between the probabilities of detection of the four species and both water temperature and the days covariate (Table 4).

## 4 DISCUSSION

River floodplains often support diverse amphibian communities because of highly variable influences of hydrology and habitat structure (Littlefair et al., 2021; Ocock et al., 2016; Tockner et al., 1998). We found a positive relationship between the mean breeding abundance of four amphibian species and hydrological isolation from the main channel of the Danube River. Amphibian diversity declined with hydrological connectivity in another floodplain system upstream of the study area (Tockner et al., 1998). Some studies suggest that this pattern is caused by fish predation in hydrologically connected wetlands (Amoros & Bornette, 2002; Ward et al., 1999), yet we found no relationship between the number of large predatory fish species at a site and amphibian abundance, although there was a strong correlation between large fish species richness and connectivity with the main river channel. We also found higher amphibian abundance at sites surrounded by high forest cover highlighting the importance of landscape composition for amphibian communities in floodplains.

### 4.1 Hydrology

Several authors have found that amphibian diversity increases with decreasing connectivity of floodplain waterbodies (Morand & Joly, 1995; Tockner et al., 1998). For example, in an Austrian Danube riverscape most fish species were recorded in the more connected waterbodies, whereas amphibian species richness peaked in disconnected waterbodies (Tockner et al., 1998). We found that mean abundance for both the metacommunity and egg masses and larvae of four species increased with decreasing connectivity of waterbodies with the main river channel. Most authors attribute this relationship to fish predation, which is expected to increase with hydrological connectivity to the river and water permanency (Amoros & Bornette, 2002; McGinness et al., 2014; Tockner et al., 2006). However, we found no support for models that included the number of large predatory fish species at sites. We detected large predatory fish (e.g., *Esox lucius*) even at the most isolated waterbodies (both permanent and ephemeral) where amphibian abundance also was comparatively high. Based on model selection, it therefore appears likely that amphibian abundance in the study area was regulated more by hydrology than the diversity of large predatory fish at a site. Likewise, Henning and Schirato (2006) found that hydroperiod affected amphibian abundances in floodplain wetlands while fish were present in both temporary and permanent waterbodies.

The abundance of *R. dalmatina* increased sharply with waterbody isolation, although spawn was detected in both permanent and ephemeral waterbodies. Studies suggest that *R. dalmatina* is more common and reproductively more successful in ephemeral habitats than other species (Hartel et al., 2011), and in other floodplains, *R. dalmatina* occurs primarily in isolated ponds in riparian forest (Tockner et al., 2006). However, *R. dalmatina* also spawns in permanent waterbodies in disconnected floodplains (Baumgartner et al., 1997). We detected *R. dalmatina* larvae in permanent ponds with fish, and this species' larvae may co‐exist with predatory fish because of behavioural adaptations (Teplitsky et al., 2003) and structural complexity in ponds (Hartel et al., 2007, 2009), although models containing habitat structure were not supported in our analysis. Nonetheless, *R. dalmatina* larvae were only detected at four of the 20 sites where egg masses were detected, which may be attributed to some larvae reaching metamorphosis and leaving the water before Survey 2, 41–49 days later (time to metamorphosis under laboratory conditions is ~4 weeks; Iosob & Prisecaru, 2014). However, there also is the possibility that some level of reproductive failure occurred at oviposition sites, possibly as a result of predation or desiccation at ephemeral sites, or because of low detectability of larvae.

Likewise, the mean abundance of *H. arborea* larvae increased at hydrologically disconnected waterbodies, but at predominantly shallow ephemeral sites or where few large predatory fish species were detected, possibly because of its vulnerability to fish predation (Teplitsky et al., 2003). We also found a strong relationship between the abundance of *Pelophylax* spp. larvae and hydrological isolation, but larvae were predominantly detected at deeper permanent waterbodies.

Conversely, the abundance of *B. bufo* increased greatly with increasing waterbody connectivity. Larvae of *B. bufo* often co‐exist with predatory fish because they are toxic and therefore unpalatable or because larvae form large schools as a defensive mechanism (Kloskowski et al., 2020), and so this species often prefers permanent waterbodies (Hartel et al., 2011). Moreover, *B. bufo* is an *r*‐selected species which breeds explosively to coincide with favourable hydrological conditions and has a short time to metamorphosis, which allows it to exploit dynamic floodplain habitats (Morand & Joly, 1995; Tockner et al., 2006). *Rana dalmatina* also is an explosive early breeding species and shares similar reproductive traits (Joly & Morand, 1994), and thus differences between these two species in breeding habitat cannot be attributed to life history.

Amphibian abundance in floodplain wetlands is often tied to natural flooding (Ocock et al., 2016). The height of the Danube River peaked in July 2021, flooding less connected sites that were dry during the first survey in March. The filling of ephemeral sites stimulated breeding activity in several species and larvae were subsequently detected. Hence, amphibian reproductive activity in the study area was strongly tied to hydrological conditions. However, no amphibian eggs or larvae were detected in the main channel of the Danube River during any surveys. In other floodplains amphibians have been observed to occupy the entire hydrodynamic gradient, except the main river channel (Littlefair et al., 2021; Tockner et al., 2006).

Despite our sampling occurring before and immediately after a major flood event, we assessed patterns in amphibian abundance over one breeding season which may limit our ability to make inferences on the response of amphibian metacommunities to flooding and hydroperiod over time. Riverine floodplains are highly dynamic ecosystems spatially and temporally (Amoros & Bornette, 2002; Ward et al., 1999). Future studies should therefore be conducted over longer temporal scales to determine community responses across different flooding and hydrological conditions (e.g., Littlefair et al., 2021; Sarker et al., 2022).

### 4.2 Landscape composition and metacommunity theory

There was a positive relationship between amphibian abundance and the proportion of forest cover within a 500‐m radius surrounding a site, and conversely a negative relationship with agricultural land. Other studies reported a similar association with forest cover for anuran species in European landscapes (Hartel et al., 2010; Van Buskirk, 2005; Zanini et al., 2008) or negative associations with agricultural land (Cayuela et al., 2015). However, there was no strong association between landscape composition and the mean estimated abundance of *B. bufo* or *H. arborea* larvae.

These interspecific differences suggest that habitat requirements of the terrestrial life stages of some species (e.g., *R. dalmatina*) include forested areas required as foraging, shelter and overwintering sites, in addition to providing suitable habitat for dispersal, while other species (e.g., *H. arborea*) can occupy a range of natural and modified habitats at the landscape scale. The positive relationship between the breeding abundance of the *Pelophylax* spp. and forest cover is surprising because Van Buskirk (2005) reported a negative effect of forest cover on the abundance of *Pelophylax* spp. larvae, although at a greater spatial scale (1000‐m radius) than in our study (500‐m radius).

There was no support for the model containing the nearest neighbour distance and there was only a small relationship between mean community abundance and landscape composition. The most influential covariate on amphibian breeding abundance was a local‐scale variable, with strong evidence of species‐sorting along the hydroperiod gradient. Hence, we assume that the mass‐effect perspective of metacommunity theory was a weaker depiction of amphibian communities in the study area, and that abundance was driven more by covariates at the local rather than at the landscape spatial scale. A similar result was obtained in a study in southern Hungary, where there was a significant effect of hydroperiod on amphibian community assembly and a weaker spatial effect (Péntek et al., 2017).

Metapopulation theory posits that abundance increases with habitat patch size (e.g., Hanski, 1994). However, we found a stronger effect of hydrology than the effect of waterbody area on mean community abundance. Likewise, in a floodplain along the Austrian Danube, hydrological connectivity overrode the effect of surface area on amphibian species richness (Tockner et al., 1999).

In our study, at the landscape scale there was a very low proportion cover of urban land within a 500‐m radius of waterbodies (mean 0.018, *SD* 0.051) with few obvious artificial barriers to amphibian dispersal, although the main river channel comprised a significant natural barrier (see Covarrubias et al., 2021). There was a high proportion of forest cover surrounding waterbodies (mean 0.735, *SD* 0.247) which is generally favourable for amphibian movement (Joly, 2019). The waterbodies that we sampled also were surrounded by other unsampled wetlands and channels in the intervening landscape, and thus were well‐connected for amphibian dispersal. A greater proportion of wet areas and forest in floodplain landscapes may facilitate species' occupancy and colonisation because they increase landscape permeability (Holgerson et al., 2019). Hence, the high degree of terrestrial connectivity and landscape heterogeneity in the study area is likely to have been responsible for the smaller effect of landscape variables on community abundance. This result also highlights the relative intactness of the floodplain in this section of the Middle Danube compared to other reaches which are substantially modified.

### 4.3 Conservation implications

Despite the limitations in making inferences on floodplain metacommunity patterns based on egg mass and larval abundance over one breeding season, our results suggest that to increase the abundance of amphibian communities we need to provide hydrologically disconnected waterbodies, especially in modified floodplains where these waterbody types may be scarce. The construction of new off‐channel wetlands is a viable strategy for adding habitats to altered floodplain landscapes (Holgerson et al., 2019). This could involve simple management interventions such as scraping shallow depressions to create ephemeral wetlands. Furthermore, protection or restoration of floodplain forests would provide important terrestrial habitat. The similarities in patterns of community abundance that we observed relative to hydrology should guide management actions for amphibians in programmes that aim to restore hydrological connectivity between floodplains and river channels.

## AUTHOR CONTRIBUTION

Conceptualisation: AJH, IC, TE. Developing methods: AJH, IC, TE. Data analysis: AJH. Conducting the research, data interpretation, writing: AJH, IC, BG, PS, ZS, BP, TE.

## CONFLICT OF INTEREST STATEMENT

The authors declare there is no conflict of interest surrounding the research undertaken in this study, funding agencies, authors' affiliations or any other potential sources of conflict.

## Supporting information


Table S1.
Table S2.
Table S3.
Table S4.
Table S5.
Table S6.
Table S7.
Table S8.
Table S9.
Table S10.

## Data Availability

Data will be made available upon reseasonable request from the first author.
