# Supplementary material for: Hydrology is a major influence on amphibian abundance in a large European floodplain
Source: Freshw Biol. 2023 May 17;68(8):1303–18. doi: 10.1111/fwb.14104 (PMC10952816; doi:10.1111/fwb.14104)
Supplement: Supplementary file 1 — Table S1. Table S2. Table S3. Table S4. Table S5. Table S6. Table S7. Table S8. Table S9. Table S10. [file FWB-68-1303-s001.docx]

ELECTRONIC SUPPLEMENTARY MATERIAL

**Hydrology is a major influence on amphibian abundance in a large European floodplain**

Freshwater Biology

Andrew J. Hamer^1,2^, István Czeglédi^1,3^, Blanka Gál^1,3^, Péter Sály^2,4^, Zoltán Szalóky^2,4^, Bálint Preiszner^1,3^, Tibor Erős^1,3^

^1^ Balaton Limnological Research Institute, Eötvös Loránd Research Network (ELKH), Klebelsberg K. u. 3, 8237 Tihany, Hungary

^2^ Institute of Aquatic Ecology, Centre for Ecological Research, Karolina u. 29, 1113 Budapest, Hungary

^3^ National Laboratory for Water Science and Water Security, Balaton Limnological Research Institute, Tihany, Hungary

^4^ National Laboratory for Water Science and Water Security, Water Ecological Institute, Budapest, Hungary

Email: [a.hamer@unimelb.edu.au](mailto:a.hamer@unimelb.edu.au)

ORCID ID: https://orcid.org/0000-0001-6031-7841

**Table S1** Correlation coefficients (Pearson’s *r*) among nine habitat structure variables, area and large fish species richness recorded at 30 waterbodies in the Middle-Danube floodplain, southern Hungary.

|  | Area | Fish | Emerge | Submerge | Float | Algae | Trees | Woody | Fringe | Canopy |
| --- | --- | --- | --- | --- | --- | --- | --- | --- | --- | --- |
| Fish | 0.282 |  |  |  |  |  |  |  |  |  |
| Emerge | –0.099 | –0.131 |  |  |  |  |  |  |  |  |
| Submerge | –0.213 | –0.527 | –0.172 |  |  |  |  |  |  |  |
| Float | –0.122 | –0.298 | 0.147 | –0.102 |  |  |  |  |  |  |
| Algae | –0.192 | –0.465 | –0.083 | 0.322 | 0.138 |  |  |  |  |  |
| Trees | –0.103 | 0.136 | 0.556 | –0.233 | –0.098 | –0.182 |  |  |  |  |
| Woody | –0.062 | 0.026 | –0.265 | 0.094 | –0.274 | –0.203 | 0.234 |  |  |  |
| Fringe | –0.137 | –0.233 | **0.670** | –0.219 | 0.399 | 0.087 | 0.166 | –0.437 |  |  |
| Canopy | –0.093 | 0.356 | –0.153 | –0.294 | –0.228 | –0.242 | 0.151 | 0.445 | –0.472 |  |
| Riprap | 0.592 | 0.304 | –0.174 | –0.087 | –0.113 | –0.151 | –0.124 | –0.011 | –0.101 | –0.073 |

Area = sampled waterbody area; Fish = number of species of large predatory fish; Emerge = % cover of emergent vegetation; Submerge = % cover of submerged vegetation; Float = % cover of floating vegetation; Algae = % cover of surface algae; Trees = % of shoreline perimeter covered by trees and other woody vegetation; Woody = % cover of woody debris; Fringe = % of shoreline perimeter covered by herbaceous vegetation/ macrophytes; Canopy = % canopy cover; Riprap = % of shoreline perimeter covered by concrete riprap

Coefficients in bold denotes a strong correlation (*r* >0.6)

**Table S2** Correlation coefficients (Pearson’s *r*) among six water chemistry variables, area and large fish species richness recorded at 30 waterbodies in the Middle-Danube floodplain, southern Hungary.

|  | Area | Fish | pH | EC | Sal | TDS | DO |
| --- | --- | --- | --- | --- | --- | --- | --- |
| Fish | 0.282 |  |  |  |  |  |  |
| pH | 0.175 | 0.194 |  |  |  |  |  |
| EC | –0.172 | –0.431 | –0.367 |  |  |  |  |
| Sal | –0.188 | –0.329 | –0.320 | **0.808** |  |  |  |
| TDS | 0.161 | –0.219 | –0.140 | **0.798** | **0.634** |  |  |
| DO | –0.060 | 0.127 | **0.690** | –0.095 | 0.035 | –0.038 |  |
| Temp | –0.117 | –0.526 | –0.003 | 0.539 | 0.375 | 0.501 | 0.049 |

Area = sampled waterbody area; Fish = number of species of large predatory fish; EC = electrical conductivity (µS/cm @ 25℃); Sal = salinity (PSU); TDS = Total Dissolved Solids (mg/L); DO = Dissolved Oxygen (mg/L); Temp = water temperature (℃)

Coefficients in bold denotes a strong correlation (*r* >0.6)

**Table S3** Correlation coefficients among three hydrology variables, area and large fish species richness recorded at 30 waterbodies in the Middle-Danube floodplain, southern Hungary. Continuous variables were correlated using Pearson correlations (*r*) whereas Permanence (categorical variable) was assessed using a Spearman rank correlation (*r*_s_).

|  | Area | Fish | Permanence | Depth |
| --- | --- | --- | --- | --- |
| Fish | 0.282 |  |  |  |
| Permanence | 0.222 | 0.465 |  |  |
| Depth | **0.915** | 0.469 | 0.462 |  |
| Connected | 0.546 | **0.703** | 0.290 | **0.651** |

Area = sampled waterbody area; Fish = number of species of large predatory fish; Permanence = permanent (1) or ephemeral (0); Depth = water depth; Connected = % of days connected to the main channel

Coefficients in bold denotes a strong correlation (*r* >0.6)

**Table S4** Correlation coefficients (Pearson’s *r*) among five landscape variables, area and large fish species richness recorded at 30 waterbodies in the Middle-Danube floodplain, southern Hungary.

|  | Area | Fish | Agri | Forest | Pasture | Urban |
| --- | --- | --- | --- | --- | --- | --- |
| Fish | 0.282 |  |  |  |  |  |
| Agri | 0.139 | 0.327 |  |  |  |  |
| Forest | –0.173 | –0.549 | **–0.782** |  |  |  |
| Pasture | 0.184 | 0.459 | 0.341 | **–0.625** |  |  |
| Urban | **0.790** | 0.323 | 0.238 | –0.357 | 0.360 |  |
| Water | –0.199 | 0.366 | 0.001 | –0.567 | 0.351 | –0.106 |

Area = sampled waterbody area; Fish = number of species of large predatory fish; Agri = % of agricultural land within a 500-m radius of a site; Agri = % of agricultural land within a 500-m radius of a site; Forest = % of forested/ semi-natural land within a 500-m radius of a site; Pasture = % of pastural land within a 500-m radius of a site; Urban = % of urban land within a 500-m radius of a site; Water = % of water/ wetland within a 500-m radius of a site

Coefficients in bold denotes a strong correlation (|*r*| >0.6)

**Table S5.** Correlation coefficients (Pearson’s *r*) among principal component scores (Landscape, Habitat, Water, Hydrology), sampled waterbody area (Area), nearest neighbour distance (Distance) and large fish species richness (Fish) recorded at 30 waterbodies in the Middle-Danube floodplain, southern Hungary.

|  | Landscape | Habitat | Water | Hydrology | Area | Distance |
| --- | --- | --- | --- | --- | --- | --- |
| Habitat | –0.447 |  |  |  |  |  |
| Water | –0.147 | 0.478 |  |  |  |  |
| Hydrology | –0.518 | 0.453 | 0.496 |  |  |  |
| Area | –0.007 | 0.084 | –0.156 | –0.410 |  |  |
| Distance | 0.371 | 0.028 | 0.022 | –0.216 | 0.288 |  |
| Fish | 0.573 | –0.297 | –0.442 | **–0.704** | 0.313 | 0.451 |

see Table 2 for an explanation of principal component scores

Coefficients in bold denotes a strong correlation (|*r*| >0.6)

**Table S6.** Large and small predatory fish species detected at 30 waterbodies in the Middle-Danube floodplain, southern Hungary.

| Species | No. of sites | Proportion of sites |
| --- | --- | --- |
| LARGE |  |  |
| *Acipenser ruthenus* | 9 | 0.30 |
| *Ameiurus melas*† | 20 | 0.67 |
| *Anguilla anguilla* | 1 | 0.03 |
| *Esox lucius* | 23 | 0.77 |
| *Leuciscus aspius* | 21 | 0.70 |
| *Lota lota* | 1 | 0.03 |
| *Micropterus salmoides*† | 1 | 0.03 |
| *Oncorhynchus mykiss*† | 1 | 0.03 |
| *Perca fluviatilis* | 20 | 0.67 |
| *Sander sp.* | 20 | 0.67 |
| *Silurus glanis* | 17 | 0.57 |
| SMALL |  |  |
| *Gasterosteus aculeatus*† | 1 | 0.03 |
| *Lepomis gibbosus*† | 18 | 0.60 |
| *Neogobius melanostomus*† | 12 | 0.40 |
| *Perccottus glenii*† | 1 | 0.03 |
| *Ponticola kessleri*† | 9 | 0.30 |
| *Pseudorasbora parva*† | 25 | 0.83 |

† invasive species in Hungary

**Table S7** Principal component scores of three hydrology variables recorded at 30 waterbodies in the Middle-Danube floodplain, southern Hungary.

| Variable | PC1 | PC2 | PC3 |
| --- | --- | --- | --- |
| Permanence | *–0.441* | **0.892** | –0.101 |
| Depth | **–0.624** | –0.385 | **–0.680** |
| Connected | **–0.645** | –0.237 | **0.726** |
| Proportion variance explained | 0.619 | 0.266 | 0.115 |

See Table S3 for a description of the variables

Coefficients in italics and underlined denotes a moderate correlation (|*r*| >0.4); coefficients in bold denotes a strong correlation (|*r*| >0.6)

**Table S8** Principal component scores of nine habitat structure variables recorded at 30 waterbodies in the Middle-Danube floodplain, southern Hungary.

| Variable | PC1 | PC2 | PC3 | PC4 | PC5 | PC6 | PC7 | PC8 | PC9 |
| --- | --- | --- | --- | --- | --- | --- | --- | --- | --- |
| Emerge | *0.445* | –0.374 | 0.184 | –0.219 | 0.059 | –0.012 | –0.349 | –0.312 | *0.598* |
| Submerge | –0.088 | *0.430* | *0.469* | –0.380 | –0.304 | –0.213 | *–0.462* | –0.142 | –0.267 |
| Float | 0.342 | 0.115 | –0.118 | *0.558* | **–0.655** | 0.147 | –0.256 | 0.158 | 0.058 |
| Algae | 0.134 | *0.416* | 0.380 | 0.095 | 0.275 | **0.745** | 0.107 | –0.059 | 0.097 |
| Trees | 0.106 | *–0.547* | 0.311 | –0.253 | –0.119 | 0.298 | –0.049 | *0.562* | –0.327 |
| Woody | *–0.418* | –0.228 | 0.273 | –0.040 | *–0.550* | 0.151 | *0.455* | –0.347 | 0.212 |
| Fringe | *0.562* | –0.098 | –0.050 | –0.050 | –0.052 | –0.006 | 0.309 | *–0.505* | *–0.562* |
| Canopy | –0.381 | –0.352 | 0.032 | *0.410* | 0.199 | 0.202 | *–0.491* | –0.387 | –0.301 |
| Riprap | –0.109 | 0.060 | **–0.643** | *–0.502* | –0.200 | *0.474* | –0.206 | –0.113 | –0.017 |
| Proportion variance explained | 0.280 | 0.218 | 0.138 | 0.111 | 0.078 | 0.073 | 0.046 | 0.041 | 0.015 |

See Table S1 for a description of the variables

Coefficients in italics and underlined denotes a moderate correlation (|*r*| >0.4); coefficients in bold denotes a strong correlation (|*r*| >0.6)

**Table S9** Principal component scores of six water chemistry variables recorded at 30 waterbodies in the Middle-Danube floodplain, southern Hungary.

| Variable | PC1 | PC2 | PC3 | PC4 | PC5 | PC6 |
| --- | --- | --- | --- | --- | --- | --- |
| pH | –0.254 | **0.643** | –0.106 | 0.359 | *0.573* | 0.229 |
| EC | *0.552* | 0.046 | 0.122 | 0.027 | –0.126 | **0.814** |
| Sal | *0.492* | 0.082 | *0.473* | –0.347 | *0.556* | –0.312 |
| TDS | *0.493* | 0.158 | 0.002 | **0.713** | –0.243 | *–0.405* |
| DO | –0.107 | **0.697** | 0.332 | –0.314 | *–0.536* | –0.089 |
| Temp | 0.366 | 0.259 | **–0.800** | –0.377 | 0.011 | –0.129 |
| Proportion variance explained | 0.497 | 0.274 | 0.112 | 0.067 | 0.031 | 0.019 |

See Table S2 for a description of the variables

Coefficients in italics and underlined denotes a moderate correlation (|*r*| >0.4); coefficients in bold denotes a strong correlation (|*r*| >0.6)

**Table S10** Principal component scores of five landscape variables recorded at 30 waterbodies in the Middle-Danube floodplain, southern Hungary.

| Variable | PC1 | PC2 | PC3 | PC4 | PC5 |
| --- | --- | --- | --- | --- | --- |
| Agri | *0.458* | –0.296 | **0.665** | –0.005 | *0.510* |
| Forest | **–0.607** | –0.090 | –0.190 | –0.202 | **0.739** |
| Pasture | *0.484* | 0.048 | –0.388 | **–0.778** | 0.092 |
| Urban | 0.301 | *–0.583* | *–0.580* | *0.458* | 0.152 |
| Water | 0.311 | **0.750** | –0.186 | 0.380 | 0.403 |
| Proportion variance explained | 0.511 | 0.235 | 0.163 | 0.091 | 0.000 |

See Table S4 for a description of the variables

Coefficients in italics and underlined denotes a moderate correlation (|*r*| >0.4); coefficients in bold denotes a strong correlation (|*r*| >0.6)
